# Estimation of Arsenic Intake from Drinking Water and Food (Raw and Cooked) in a Rural Village of Northern Chile. Urine as a Biomarker of Recent Exposure

**DOI:** 10.3390/ijerph120505614

**Published:** 2015-05-22

**Authors:** Oscar Pablo Diaz, Rafael Arcos, Yasna Tapia, Rubén Pastene, Dínoraz Velez, Vicenta Devesa, Rosa Montoro, Valeska Aguilera, Miriam Becerra

**Affiliations:** 1 Department of Biology, Faculty of Chemistry and Biology, University of Santiago of Chile, Av. Bernardo O’Higgins 3363, Santiago 9160000, Chile; 2 Ealth Service of Calama, Cobija 2188, Calama 1390000, Chile; E-Mail: arcoszabala@gmail.cl; 3 Faculty of Agricultural Science. University of Chile, Av. Santa Rosa 11315, La Pintana 8820000, Santiago, Chile; E-Mail: yasnatapiafernadez@gmail.cl; 4 Department of Chemistry of Materials, Faculty of Chemistry and Biology, University of Santiago of Chile, Av. Bernardo O’Higgins 3363, Santiago 9160000, Chile; E-Mail: ruben.pastene@usach.cl; 5 Instituto de Agroquímica y Tecnología de Alimentos, CSIC. PO Box 73, 46100 Burjassot, Valencia, Spain; E-Mails: deni@iata.csic.es (D.V.); vicenta.devesa@iata.csic.es (V.D.); rmontoro@iata.csic.es (R.M.); 6 Department of Geographical Engineering, Faculty of Engineering, University of Santiago of Chile, Av. Bernardo O’Higgins 3363, Santiago 9160000, Chile; E-Mails: valky.nadhy@gmail.cl (V.A.); miriam.becerra@gmail.com (M.B.)

**Keywords:** arsenic, endemic, area, drinking water, food, urine

## Abstract

The aim of this study was to estimate both the contribution of drinking water and food (raw and cooked) to the total (t-As) and inorganic (i-As) arsenic intake and the exposure of inhabitants of Socaire, a rural village in Chile´s Antofagasta Region, by using urine as biomarker. The i-As intake from food and water was estimated using samples collected between November 2008 and September 2009. A 24-hour dietary recall questionnaire was given to 20 participants. Drinking water, food (raw and cooked) and urine samples were collected directly from the homes where the interviewees lived. The percentage of i-As/t-As in the drinking water that contributed to the total intake was variable (26.8–92.9). Cereals and vegetables are the food groups that contain higher concentrations of i-As. All of the participants interviewed exceeded the reference intake FAO/OMS (149.8 µg∙i-As·day^−1^) by approximately nine times. The concentration of t-As in urine in each individual ranged from 78 to 459 ng·mL^−1^. Estimated As intake from drinking water and food was not associated with total urinary As concentration. The results show that both drinking water and food substantially contribute to i-As intake and an increased exposure risk to adult residents in contaminated areas.

## 1. Introduction

Arsenic (As) is distributed extensively throughout the World. The main sources of As are pesticides and fertilizers, metallurgic industrial waste from melting and mining operations, and low levels of As are commonly found in the air, soil and water [1]. Inorganic arsenic (i-As) [As(III) + As(V)] is the most toxic chemical As species present in food and drinking water. Arsenic in drinking water is generally in the form of either arsenite (As^III^), from deep anaerobic wells, or arsenate (As^V^), which predominates under aerobic conditions [2]. Arsenic has been classified by the International Agency for Research on Cancer (IARC) as a human carcinogen [3].

The general population is exposed to As principally through drinking water and food, the two primary ingestion pathways [1]. The most common sources of As pollution in the water and soil of the Antofagasta Region of Chile are its natural hydrogeological characteristics, the presence of volcanoes and mining activities [4].

Arsenic not only contaminates water but also grains, vegetables and food stuffs in general, consequently endangering human and animal health [5]. There is a potential risk to human health via the consumption of agricultural produce grown in fields irrigated with As contaminated water [6].

Epidemiological studies carried out in arsenic-endemic areas have found drinking water to be the primary source of As for humans [7,8,9]. However, some studies have shown that the levels of i-As excreted in urine are higher than expected based on the levels of this toxic in drinking water alone [10,11,12]. As a result, food sources are thought to be a considerable source of As intake, and studies of the total arsenic (t-As) levels in food obtained from arsenic-endemic areas have increased in recent years [2,13,14]. Epidemiological studies of low-level arsenic in drinking water (e.g., generally ≤ 100 µg·L^−1^) in populations from the U.S., Finland, Argentina, and northeastern Taiwan indicate that, although exposure in these areas exceeds background rates, this low level exposure is not associated with a significantly increased risk of bladder cancer. However, at higher concentrations the risks increase and the most common symptoms related to such exposure are skin lesions, melanosis, conjunctivitis, keratosis and hyperkeratosis, while other more extreme health effects have also been reported [15,16].

In arsenic-endemic areas, the high levels of As in water used for cooking purposes is an additional source of contamination, primarily in the form of i-As. Other authors have suggested that As-contaminated water used in food preparation is the origin of the high t-As levels detected in cooked food [14,17,18]. Food and drinking water together typically account for 99% of the total human As intake [19].

Therefore, daily As intake, primarily in the form of i-As, from meals and drinking water, is a fundamental parameter for health evaluations. This parameter also provides an additional check on the effectiveness of regulations on the As levels in water and food [19].

There are several potential biomarkers for As exposure. Urine biomarkers are preferred, as they are relative easy to collect and, because the majority of absorbed As is eliminated via urine (including various metabolites). Current analytical techniques allow for As speciation in urine samples, but not in hair and nails samples [20].

After As intake, a certain proportion of the compound is accumulated in different parts of the body. However, approximately 40%–60% of the ingested form is eliminated through urine within 1–2 days. Thus, the measurement of urinary As levels is considered the most reliable indicator of recent exposure to As [21].

The present work focuses on the Antofagasta Region, an area of 126,049 km^2^ located in northern Chile where there are high As levels in the environment. This area is extremely arid and contains the Atacama Desert, which is one of the most arid regions of the world. The major cities in the Antofagasta Region now have water treatment plants. Drinking water from this region contains the maximum As permitted by the FAO/OMS (10 µg·As·L^−1^). However, most of the small towns do not have treatment plants, and consequently, the population consumes water containing higher As levels than the maximum amount permitted by Chilean and World Health Organization (WHO) legislation (10 µg∙As·L^−1^). Socaire is one such town, where a study carried out by Martínez *et al.* [22], measured high levels of As in drinking water (0.26 mg·L^−1^). In addition, agricultural activity in these small towns with high levels of As contamination in the soil, air, and irrigation water may have negative effects on food safety [4,23].

The aim of the present study is to evaluate the contribution of drinking water and food, both raw and cooked to the total and inorganic arsenic intake by using urine as a biomarker to estimate the As exposure of the inhabitants of Socaire, a rural village in Chile’s Antofagasta Region. This work uses chemical analysis of consumed foods and water to estimate the i-As intake in arsenic endemic-areas on the basis of experimentally obtained i-As levels rather than extrapolating from assumed t-As levels present in food.

## 2. Methods

### 2.1. Study Area

**Figure 1:**
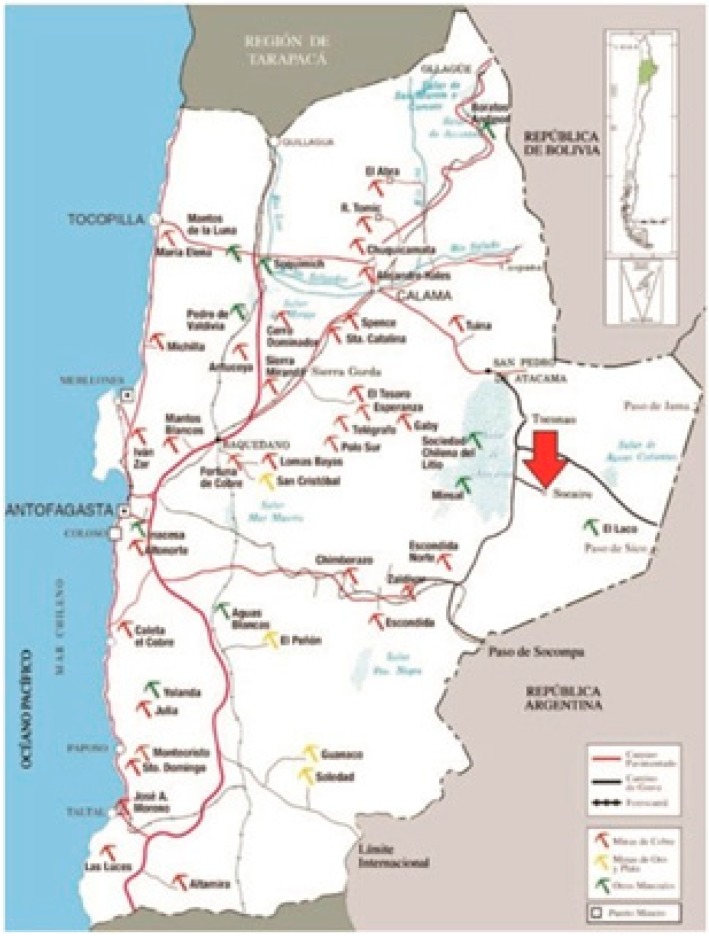
A map showing the location of the study area (Socaire village, Chile).

The study presented in this paper was carried out between November 2008 and September 2009, in the agricultural village of Socaire (23°36′00′′ S and 67°50′60′′ W), located 180 km east of Calama, the city with the second largest population in the region (Figure 1). The village of Socaire had 255 residents at the time of the study, however only 137 (66 men and 71 women) of them belong the district of Socaire, a small locality where this study was carried out. The district of Socaire is an indigenous community in the Andes, primarily composed of ethnic Atacameños. The Atacameño people have lived in northern Chile and Argentina, areas with high As ground water levels, for thousands of years. Adults comprise 66% of this population (age: 39.5 ± 1.42), and agriculture is the main economic activity in Socaire.

### 2.2. Food Questionnaire

A 24-hour dietary recall questionnaire was given to 20 residents (five men and 15 women) in the township of Socaire; interviews were conducted in the participant’s homes. The questionnaire is useful when needing information about water sources used for cooking and drinking water in the home (kitchen tap water, bottled water, *etc.*) and frequency of use of each water source, portion size, prompts using food models and responses from interviewers, including amount of consumed, specifics of preparation, and time of consumption. This instrument is a highly regarded method to estimate arsenic intake from drinking water and food and urinary arsenic concentrations [24]. The questionnaire asked for information about the type and quantity of water consumed, foods ingested and how the foods were prepared for consumption (raw or cooked), demographic characteristics (such as age, sex, place of residence and occupation), information on potential exposures sources (such as drinking water sources and smoking patterns), and health effects (Table 1). The health status of all interviewees was classified as acceptable (A), regular (R) and deficient (D). An *acceptable* health status was assigned to an interviewee who did not complain of any clinical symptoms; *regular* to interviewees who declared some clinical symptoms, including abdominal pain; and *deficient* to interviewees who declared several clinical symptoms, including cardiovascular disease and/or hypertension [13]. The Regional Ministerial Health Secretariat for Chile’s Antofagasta Region approved the design of the questionnaire and the methods used to perform the dietary study; additionally these methods were applied in a previous study [14]. Professionals from the Health Service of San Pedro de Atacama (Antofagasta Region) and our research group administered the questionnaire. Body weight was assumed to be 70 kg and the mean age was 53 years. All participants had been living in the village for over five years at the time of our interviews and they are representative of their community (Table 1). Randomly selected households were identified through door-to-door contact. One person was selected randomly among household adult participants and identified as the primary respondent [24]. Children and young adults were not included because they were absent at the time of our interviews.

**Table 1:** Main characteristics of the study population.

No.	Age (years)	Sex	Employment	Period of Residence (years)	Health Status	Health Effects
1	31	F	unemployed	> 15	A	not declared
2	39	F	commerce	> 5	D	gastrointestinal disease, skin hyperpigmentation
3	40	F	unemployed	> 5	R	gastrointestinal and respiratory diseases
4	41	F	commerce	> 15	R	skin hyperpigmentation
5	41	F	services	> 5	R	gastrointestinal disease
6	47	F	unemployed	< 5	R	gastrointestinal disease
7	49	F	unemployed	> 15	D	gastrointestinal disease, skin hyperpigmentation
8	51	F	unemployed	> 15	R	gastrointestinal and vascular diseases
9	54	F	farming/commerce	> 15	R	gastrointestinal disease
10	56	F	farming	> 15	A	not declared
11	57	F	commerce	> 15	R	gastrointestinal disease
12	62	F	unemployed	> 5	R	nutritionaldisease (obesity)
13	64	F	unemployed	> 15	D	vascular disease
14	73	F	farming	> 15	D	skin hyperpigmentation, tumor
15	76	F	unemployed	> 15	A	not declared
16	46	M	mining	> 15	R	respiratory and vascular diseases
17	52	M	commerce	> 5	A	not declared
18	52	M	commerce	> 5	A	not declared
19	58	M	farming	> 15	D	gastrointestinal disease, skin hyperpigmentation
20	68	M	mining	> 15	A	not declared

Notes: M: male; F: female; A: acceptable; R: regular; D: deficient.

### 2.3. Water and Food Samples

Samples of drinking water and food (raw and cooked) consumed on the day of our visit, were collected directly from interviewees’ homes. The source of all collected drinking water samples was the Quepes watershed, located near the district of Socaire. Drinking water samples were collected from the kitchen tap (or other designated drinking water sources, not including bottled water), after allowing the water to run through the pipes for several seconds, in acid-washed polyethylene bottles. The bottle was filled to the top and labeled. The water samples were placed in a refrigerated container for transport. Control samples were obtained from three homes located in Calama, one of the largest cities in the Antofagasta Region which now has a water treatment plant. To preserve the As in water samples, 0.5 mL of HCl (0.01 mol·L^−1^) was added to each sample. The water samples were transferred to the laboratory and were stored at 4 °C until As analysis.

Most food products sampled were grown in the local community of Socaire and were divided into three groups: cereals, vegetables and meats. Once food samples were collected a mixture of these foods was placed in an aluminum box with a capacity of 500 g, frozen at −20 °C and then freeze-dried. The lyophilized samples were ground in a domestic apparatus, and the resulting powder was vacuum packed and stored at 4 °C until As analysis.

### 2.4. Collection and Preparation of Urine Samples

Participants from Socaire were supplied a polyethylene bottle with instructions for urine collection prior to the day of their interview. Participants were asked to provide a first morning urine sample directly into polyethylene bottles. Concentrated HCl (Probus PA, d = 1.19; 1 mL HCl to 100 mL urine) was added to prevent bacterial growth and to preserve As content. During transport the samples were kept on wet ice, upon arrival to the laboratory the samples were then frozen at −20 °C and stored until analysis. Prior to analysis, the samples were filtered through a 0.45 µm syringe filter and diluted up to 5-fold with 2% HNO_3_ for t-As determination. Control samples were obtained from three individual adults. Control 1: Female, 48 years old, transporter; Control 2: Male, 58 years old, professional; Control 3: Male, 70 years old, professional.

### 2.5. Analysis of Samples for Arsenic

The quantification of t-As and i-As was performed with a Perkin Elmer (PE) model 3300 atomic absorption spectrometer (AAS) equipped with an autosampler (PE AS 90) and a hydride generation flow injection system (PE FIAS-400). Deionized water (18.2 M·Ω·cm) obtained with a Milli-Q water system (Millipore Inc., Millipore Ibérica, Madrid, Spain) was used for the preparation of reagents and standards. Calibration standard solutions of As(III) were prepared from a reduced commercial standard solution (1000 mg·L^−1^) of As(V) (Merck, Barcelona, Spain). A mixture containing 5% (*w/v*) KI and 5% (*w/v*) ascorbic acid was used as a reducing solution.

The t-As levels in food and drinking water samples were measured using the procedures described by Díaz *et al.* [14] and Díaz *et al.* [25], respectively. Analysis of the arsenic levels in urine was measured using the procedures followed by Pandey *et al.* [26]. Total urinary As measures both inorganic and organic components of As. Instead, determination of urinary As by hydride generation-atomic absorption spectrophotometry technique (HG-AAS) enables measurement of inorganic As (As^III^ and As^V^) together with its biotransformed species, MMA and DMA. For a QA/QC analysis of the analytical data, two reference materials certified for t-As levels (National Institute of Standards and Technology) were employed: tomato leaves (SRM 1573) and rice flour (SRM 1568a). The following analytical characteristics were obtained using this method: detection limit = 0.006 µg·g^−1^ wet weight (ww), precision = 2%; the accuracy for the tomato leaves was 0.28 ± 0.02 µg·g^−1^ dry weight (dw), certified value = 0.27 ± 0.05 µg·g^−1^ dw; the accuracy for the rice flour was 0.29 ± 0.03 µg·g^−1^ dw; certified value = 0.29 ± 0.03 µg·g^−1^ dw.

The i-As levels in food samples were measured using the methods described by Muñoz *et al*. [23]. Deionized water (4.1 mL) and concentrated HCl (18.4 mL) were added to 0.5 g of freeze-dried sample. The mixture was left overnight. After reduction by HBr and hydrazine sulfate, the inorganic arsenic was extracted into chloroform and back-extracted into 1 mol∙L^−1^ HCl. The back-extraction phase was dry-ashed and the i-As was quantified by flow injection-hydride generation atomic absorption spectrometry (FI-HG-AAS).

The following analytical characteristics were obtained for this method: detection limit = 0.003 µg∙g^−1^ ww, precision = 4%, As(III) recovery = 99%, As(V) recovery = 96%. A quality criterion between the i-As ranges found in the rice flour sample and those previously reported in a study conducted in our laboratory (0.110 ± 0.03 µg∙g^−1^ dw) was adopted for this study. This value was similar to the only available reference for i-As in rice flour (0.092 µg∙g^−1^ dw) obtained by a trifluoroacetic acid extraction—ion chromatography—inductively coupled plasma mass spectrometry technique [27]. Throughout the experiment, the quality assurance/quality control of the t-As levels measured was checked by analyzing urine against certified reference material (MedisafeRMetalle U, Level 1, Medichem, LGC Standards) for each batch of samples (certified value = 0.50 µg∙mL^−1^).

### 2.6. Statistical Analyses

All statistical tests were carried out using the SPSS (Statistical Product and Service Solutions) 13.0 software package. We calculated mean values, medians, standard deviations, precision of the methods and ranges. Additionally, we applied a correlation test with urinary arsenic as the outcome variable and arsenic intake from water and food as the predictor variables.

## 3. Results

### 3.1. Health Status of the Study Population

The information collected regarding the health condition of the study population is shown in Table 1. The majority of the interviewees were women who present a *regular* or a *deficient* health condition; and the main diseases reported included gastrointestinal track problems and/or other anomalies such as skin hyperpigmentation or vascular disease.

### 3.2. Water and Food Arsenic Concentrations

#### 3.2.1. Arsenic Concentrations in Drinking Water

The concentrations of t-As found in drinking water (µg·L^−1^) and the total liquid (L day^-1^) consumed by each interviewee are listed in Table 2. For intake calculations, the United States Environmental Protection Agency (US EPA) recommends an average water intake of 1.4 L·day^−1^ and a weight of 70 kg for an adult [28], which is similar to the mean liquid ingested by the study population. The t-As concentration in the drinking water ranged from 247 µg·L^−1^ to 357 µg·L^−1^, with an average of 276.5 ± 31.9 µg·L^−1^. These As values were much higher than those of the control samples from Calama.

#### 3.2.2. Arsenic Concentrations in Food and Food Intake for Each Interviewee

The proportion (%) of food consumed from each of the three different categories and the specific subtypes of food consumed by the interviewees are shown in Table 3. All vegetable products, some of the cereal products (such as quinoa) and the lamb meat were produced locally in the community of Socaire. The principal foods consumed by the inhabitants consisted of cereals (48.5%) and vegetables (28.3%). The most commonly consumed food subtype was wheat (39.6%), followed by lettuce (39.3%). Data regarding the quantity of food ingested (g·day^−1^) and the total and inorganic As concentrations in food for each interviewee are shown in Table 4.

**Table 2:** Total arsenic concentration in the drinking water (µg·L^−1^) and total liquid consumed (L·day^−1^) for each interviewee.

Interviewee	t-As (µg·L^−1^)	Liquid ^a^ (L·day^−1^)
1	271	1.4
2	253	2.2
3	351	1.0
4	259	1.2
5	273	1.0
6	271	1.4
7	289	1.0
8	247	0.5
9	286	1.5
10	273	1.4
11	267	1.9
12	258	1.0
13	249	1.2
14	326	1.2
15	267	1.0
16	357	1.4
17	260	1.2
18	249	1.4
19	265	1.0
20	259	1.6
Range	247–357	0.5–2.2
Mean ± SD	276.5 ± 31.9	1.3 ± 0.1

Notes: Controls: 6.7 ± 0.3 (*n* = 3), t-As (µg∙L^−1^). ^a^ Including drinking water, hot beverages, soup and juice.

**Table 3:** Summary of the main food types consumed by the inhabitants of Socaire. Listed as the percentage (%) of food consumed by category (cereal, vegetable or meat) and then by specific food subtype.

Food Category/Subtype	Percentage
Cereals	48.5
Wheat	39.6
Rice	32.9
Quinoa	27.5
Vegetables	28.3
Lettuce	39.3
Carrots	32.1
Tomatoes	28.6
Meats	23.2
Red Meat	39.1
Lamb	34.8
Chicken	26.1

**Table 4:** Mixture food intake (g·day^−1^) for each interviewee by category, and specific food subtype. Arsenic concentration in the mixture.

Interviewee	Category and Subtype of Food									Arsenic Concentration		
	Cereals (Cooked)			Vegetables (Raw)			Meats (Cooked)			t-As	i-As	i-As/t-As
	Wheat	Rice	Quinoa ^1^	Carrots ^1^	Lettuce ^1^	Tomatoes ^1^	Red Meat	Lamb	Chicken	(μg·g^-1^ ww)		(%)
1	150	120	nc	100	nc	nc	nc	100	nc	0.11	0.09	81.8
2	340	120	nc	nc	nc	nc	100	200	nc	0.11	0.10	90.9
3	400	300	300	nc	100	nc	nc	nc	nc	0.24	0.21	87.5
4	300	120	200	nc	100	nc	100	nc	nc	0.22	0.13	59.1
5	100	nc	300	nc	50	150	100	nc	nc	0.19	0.17	89.5
6	220	nc	200	nc	nc	100	nc	200	nc	0.52	0.38	73.1
7	500	300	300	nc	nc	nc	nc	nc	nc	0.12	0.11	91.7
8	100	120	nc	80	80	nc	100	nc	nc	0.13	0.10	76.9
9	120	120	300	80	50	nc	150	nc	nc	0.72	0.71	98.6
10	470	120	200	nc	nc	60	150	150	nc	0.62	0.56	90.3
11	340	120	nc	80	100	nc	100	100	nc	0.15	0.14	93.3
12	100	nc	nc	nc	nc	150	200	nc	nc	0.06	0.04	66.7
13	200	120	200	nc	100	100	150	nc	200	0.81	0.21	25.9
14	100	120	nc	nc	50	nc	100	nc	nc	0.33	0.25	75.8
15	220	nc	nc	80	100	nc	nc	nc	100	0.65	0.16	24.6
16	200	200	nc	50	nc	100	100	100	nc	0.52	0.32	61.5
17	200	120	nc	80	80	nc	100	nc	nc	0.14	0.09	64.3
18	130	nc	200	80	80	80	100	nc	100	1.02	0.83	81.4
19	200	120	nc	50	nc	nc	150	150	nc	0.50	0.16	32.0
20	200	nc	nc	nc	nc	nc	100	nc	nc	0.08	0.08	100
Range										0.06–1.02	0.04–0.83	24.6–100
Mean ± SD										0.34 ± 0.3	0.24 ± 0.2	73.3 ± 23.1

Notes: nc: not consumed; ^1^ grown locally.

Individual food consumption and As levels in foods consumed by the Socaire inhabitants were both highly variable. As levels fluctuated between 0.06 to 1.02 µg·g^−1^ for t-As and 0.04 to 0.83 µg·g^−1^ for i-As. The percentage of i-As with respect to t-As, varied between 24.6% and 100%. Locally produced food, specifically vegetables and quinoa, were a regular part of the participants’ diet as determined by the food questionnaire. Samples containing these foods showed higher As levels than those food samples that were not produced locally (Table 4). The fact that individuals 9, 13, and 18 were the three individuals who consumed the most locally produced foods, also have the highest levels of As confirms this observation. The As concentration could have increased if these foods were prepared using certain cooking processes. Similar findings were also described by Queirolo *et al*. [4] and Laparra *et al*. [18].

### 3.3. Estimation of Total As Intake and Recent As Exposure

#### 3.3.1 Estimation of the Total As Intake (Drinking Water + Food)

From the data provided in the 24-hour recall questionnaire administered to the inhabitants of Socaire (quantity of water, L day^-1^ and food ingested, g, ww) and the t-As and i-As concentrations found in the water and food (raw and cooked) samples analyzed (Table 2 and Table 4) we calculated the total intake of t-As and i-As for each interviewee [14]. These intakes values, expressed as µg day^-1^, are shown in Table 5. To estimate the health risks of these As intake levels, the values were compared to the Provisional Tolerable Weekly Intake (PTWI) values of 15 µg i-As/week/kg body weight recommended by the Joint FAO/WHO Expert Committee for Food Additives (JECFA) [29] and Benchmark Dose (Lower Confidence Limit) fix a range of 0.3–8 µg i-As/day/kg body weight [30]. Additionally we measured t-As concentration in urine for each interviewee to estimate recent exposure (Table 5).

**Table 5:** Dietary arsenic intake ^a^ from water (μg·day^−1^) and food (μg·day^−1^, ww), along with t-As in urine (ng·mL^−1^) for each interviewee.

Interviewee	As intake in drinking water ^b^ (μg·day^−1^)	i-As intake in food (μg·day^−1^)	Total i-As intake (drinking water+food) (μg·day^−1^)	Percentage of drinking water support (%)	t-As in urine ^c^ (ng·mL^−1^)
1	125	56.0	181.0	69.1	78
2	396	140.0	536.0	73.9	153
3	351	231.0	582.0	60.3	416
4	311	115.7	426.7	72.9	111
5	513	124.6	637.6	80.5	459
6	378	330.6	708.6	53.3	256
7	290	121.0	411.0	70.6	365
8	260	23.2	283.2	91.8	149
9	440	930.1	1370.1	32.1	292
10	380	901.6	1281.6	29.7	116
11	312	194.4	506.4	61.6	112
12	350	954.5	1304.5	26.8	272
13	299	340.2	639.2	46.8	105
14	396	140.0	536.0	73.9	122
15	267	92.8	359.8	74.2	84
16	504	358.4	862.4	58.4	294
17	378	51.3	429.3	88.1	176
18	416	32.0	448.0	92.9	85
19	265	129.6	394.6	67.2	387
20	550	119.0	669.0	82.2	347
Range	125–550	23.2–954.5	181–1370	26.8–92.9	78–459
Mean ± SD	359.1 ± 100	269.3 ± 300.3	628.4 ± 335.4	65.3 ± 19.5	219 ± 125.8
Median	364.5	134.8	536.0	68.2	164.5

Notes: ^a^ daily intake reference value for i-As: 149.8 µg i-As day^−1^ for > 20 age group; ^b^ including hot beverages, soup and juice: ^c^ t-As in urine (ng·mL^−1^) which include inorganic arsenic (As^III^ + As^V^) and organoarsenicals Controls: 24 ± 12.8 (*n* = 3).

In order to compare PTWI values with data obtained for the inhabitants of Socaire, we used the procedure carried out by Signes-Pastor et al. [31] and Anawar et al. [32] along with the following two approximations: (1) as the questionnaire administered was a 24-hour dietary recall, we expressed the PTWI as a tolerable daily intake (TDI = PTWI/7 days), with a value of 2.14 µg i-As·kg^−1^ body weight; (2) as all of the interviewees were adults, we assumed a body weight of 70 kg, making the reference intake values stated by the FAO/OMS equivalent to 149.8 µg i- As·kg^−1^ (TDI x kg body weight) and this final value was compared to the i-As intake levels estimated for the inhabitants of Socaire [14].

The inorganic arsenic intake of all interviewees showed great variability (181.0–1370.1 µg·i-As·day^−1^, mean 628.4 ± 335.4, Table 5). This was partially due to the variable consumption of drinking water. The variability was also influenced by the volume and type of foods analyzed, in particular vegetables and cereals, in which i-As prevails. Previous studies in other arsenic-endemic areas, in which the food groups considered in the intake estimate were similar to those analyzed in Chile, estimated i-As intake by extrapolation from the t-As intake, either assuming that i-As represented all of the t-As measured [17] or at least 50% of the t-As measured [28].

The percentage of i-As/t-As in the drinking water that contributed to the total intake was variable (26.8–92.9, Table 5) although several studies have concluded that the contribution of drinking water to the i-As intake is usually high [33,34].

The FAO/WHO [35] reference intake (149.8 As·kg^−1^) was exceeded by all interviewees (Table 5), particularly interviewees 9, 10, and 12 whose intake was approximately nine times greater than the reference intake, along with greater food consumption than other interviewees (Table 4). Furthermore, more than 90% of the t-As ingested by these individuals was i-As (Table 4).

#### 3.3.2. Estimation of Recent as Exposure

The concentrations of t-As in urine for each interviewee are shown in Table 5. The t-As concentration in urine ranged from 78 ng·mL^−1^ to 459 ng·mL^−1^, with an average value of 219 ± 125.8 ng·mL^−1^. These values were much higher than those obtained in the control samples from Calama (24 ± 12.8 ng·mL^−1^). These results were also much higher than the recommended values for urinary arsenic levels in non-occupational population arsenic exposure, which is less than 20 µg·t-As·L^−1^ [36]. From Table 5 it is possible to observe that individual 20, a 68-year-old man, shows a high concentration of As in urine (347 ng·mL^−1^) and that interviewee 1, a 31-year-old woman, shows the lowest (78 ng·mL^−1^). These participants also showed the highest and the lowest As intake in drinking water (550 µg·day^−1^ and 125 µg·day^−1^), respectively. It is known that there is an association between very high As intake in drinking water and As levels in urine. To test this, we was applied a correlation test with urinary arsenic as the outcome and with arsenic intake from water and food as the predictor variables. The Pearson correlation analysis indicated that the correlation is not significant at the level of *p* ≤ 0.01 for either food or water.

## 4. Discussion

The sample size and demographic characteristics of our study participants (n = 20 individuals), was similar to the studies carried out by Martínez *et al*. [22] and Loffredo *et al*. [21]. The aim of the first of these studies was to add more information on the possible genotoxic risk associated with high levels of arsenic in drinking water consumed by inhabitants living in the villages of San Pedro de Atacama (*n* = 25 individuals), Toconao (*n* = 16 individuals), Socaire (*n* = 4 individuals) and Peine (*n* = 8 individuals); the ethnicity of all participants was Atacameño. The study carried out by Loffredo *et al*. [21], included subjects from two towns located in northeastern Chile with different levels of i-As in drinking water. Residents from Toconao (*n* = 9 participants) consumed drinking water containing 21 µg·L^−1^, while those in San Pedro de Atacama (*n* = 12 participants) consumed drinking water containing 593 µg·L^−1^.

The variation in mean As concentrations of drinking water was likely due to the natural origin from which the water was collected. Indeed, it is known that variations in the arsenic levels, as well as of other elements in water, are due to variations in the natural source input from which the water was obtained [37]. These values were also much higher than the maximum limit for As levels in drinking water as established by FAO/OMS (10 µg·L^−1^) [38], demonstrating a high level of pollution in the drinking water of Socaire. The differences between Calama and Socaire are likely due to the fact that drinking water in Calama is treated in an As-abatement plant, unlike the drinking water in Socaire, which is collected from natural sources and distributed to households without any treatment. These results agree with those of Bae *et al*. [39], who measured arsenic concentrations of between 223–372 µg·L^−1^ consumed by the population of Bangladesh. A study in the native Andean region of Argentina, found an average arsenic concentration of 200 mg·L^−1^ in drinking water [10]. However, other data on As levels in drinking water sampled during two different periods in the village of Chiu Chiu (Antofagasta Region, Chile) were higher than those obtained in our study (572 µg·L^−1^) [14].

The arsenic concentrations found in food are in agreement with those obtained by Bae *et al*. [39] who measured arsenic concentrations between 0.22 and 0.38 µg·g^−1^ (ww) in foods consumed by inhabitants of Bangladesh, with the highest values measured in rice, indicating that As water levels used during the cooking process contributed to the As content. Few studies have evaluated the effects of the cooking process on the levels of t-As and As speciation in Latin America. Health risk assessments of As intake are generally based on the content of the measured raw products. However, food is often consumed after having been processed and cooked, which could alter the total As content and its chemical speciation [40].

Other studies in the native Andean region of Argentina and the village of Chiu Chiu, showed large differences in As content in food. In the first study, t-As concentrations in foods such as soup and corn were found to be 0.43 μg·g^−1^ ww and 0.42 μg·g^−1^ ww, respectively, but it is possible that the water used in cooking was the cause of such high levels [10]. In the second study, conducted by Diaz *et al*. [14], some of the highest levels of inorganic arsenic were measured in maize grains (1.42 μg·g^−1^ ww) boiled in water containing 572 μg·As·L^−1^. The As levels in the drinking water and cooked foods from As-endemic areas showed similar levels to those found in samples collected from Chile, and it is possible that the water used in cooking was the cause of the high levels observed in both studies. Diaz *et al*. [14] experimentally used distilled water for cooking and observed a decrease in t-As and i-As with respect to the raw product. The mechanisms for capturing i-As in food might be related to the incorporation of water into the food during cooking. Thus, food samples that contained rice and quinoa, both common foods in the diets of Andean populations, such as the inhabitants of Socaire, the increase in i-As may be due to the high starch content in these cereals (80%), which incorporates a large quantity of water during its gelatinization in the boiling process [18,40]. In other samples, such as the vegetables chard, pumpkin, and carrot, which retain a considerable quantity of water during boiling, there was also an increase in i-As content [23].

Our results are similar to previously published studies on the preparation and ingestion of food (raw and cooked) in arsenic-endemic areas, which have also found that cereals and vegetables were the principal dietary components [14,23,41,42]. Variations in the t-As and i-As concentrations, as well as the percentage of i-As with respect to t-As observed in food, could be explained by the type of food tested and the specific preparation procedures used. With regards to food preparation, the use of water with high As levels can have a significant impact on the As levels in food products consumed by residents. Similar findings were also described by Queirolo *et al*. [4] and Laparra *et al*. [18]. Inhabitants of Socaire, consume drinking water and food with high As levels, primarily i-As. The consumption of cereals and vegetables contribute to high As levels depending on the ingested quantity. These results suggest that the high As content in food is primarily derived from cooking water, which contains high As concentrations. The high percentages of i-As present in food consumed by the inhabitants of Socaire suggests a high risk to human health.

The As values obtained in our study are higher than those obtained by Oguri *et al*. [43], who collected diet samples from 25 subjects in Japan. The average intake of i-As for the 25 subjects was 3.8 μg·day^−1^ (2.0–57 μg·day^−1^) and an intake of 27 μg·day^−1^ was estimated from the Typical Japanese Diet. In our work, the inclusion of other foods in the sample mixture, such as meats, may have increased the final of i-As intake estimate. Several interviewees declared that they had consumed meat products, particularly lamb and beef, and to a lesser extent chicken and llama. As in the case of horticultural products, cooking could increase the i-As levels in meat products as well. Indeed, Del Razo *et al*. [41] measured high t-As levels in cooked meats (mean value = 0.34 µg·g^−1^ww). In our study, none of the interviewees declared that they had eaten fish.

Because of the paucity of previous reports regarding As intake in As-endemic areas, it is only possible to compare the results obtained from Socaire to those obtained from Mexico and India. In a study conducted by Del Razo *et al*. [41] in the Lagunera Region of Mexico, the t-As intake was estimated from the contribution of water, hot beverages and cooked foods. In an exposed population that consumed water with a mean As concentration of 0.41 µg·mL^−1^, which was higher than the concentration observed in this study, the estimated intake was six to eight times greater than the PTWI. Studies carried out by Roychowdhury *et al*. [12,17] in West Bengal, India, also provided data for t-As intake from water and raw and cooked food. Assuming that at least 50% of the t-As in food samples is i-As, the maximum intake obtained from water and food was 708 µg day in adults, which was 4.7 times greater than the TDI. This value was similar to that obtained in our study (mean value: 628.4 ± 335.4 µg/day/adult, Table 5).

Epidemiological studies on the effects to human health of oral exposure to i-As have made it possible to establish various guideline levels. An i-As intake of 10–50 μg i-As/day/kg body weight contributed to vascular problems, which might ultimately lead to necrosis and gangrene of the hands and feet [35]. Arsenicosis studies from Taiwan reported mild improvement in the neurological manifestation on stoppage of arsenic contaminated water, but increased incidence of neurological, cardiovascular, cerebrovascular, cancer of the skin, bladder and lung and metabolic disease in the formerly arsenic exposed population [26].

Because of their long-term exposure to very high levels of i-As in drinking water, the populations of some rural villages in northern Chile and other countries have been used in numerous epidemiological studies to assess the associations between As exposure and parameters such as methylation patterns or cancer [35,44,45]. One of these studies indicate that there is prevalence of skin lesions among men who have been drinking contaminated water for more than 20 years, as well as in children in these regions; similar associations have been reported regarding As drinking water concentrations in Taiwan and west Bengal, India, both areas containing populations in which extensive malnutrition has been thought to increase susceptibility [35].

Raynaud’s syndrome, which is classically described as affecting the acral parts of the body, most commonly the digits of the hand and feet, has been reported in people drinking water with high As levels in northern Chile [44].

Therefore, it appears that neither good nutrition nor many centuries of exposure protect the inhabitants of Socaire from the adverse health effects of i-As exposure, which may explain the presence of some of the symptoms described by interviewees such as skin effects, hyperpigmentation, gastrointestinal and vascular diseases.

Martinez *et al.* [22] evaluated whether or not environmental exposure to arsenic in ground water results in a significant increase in the frequency of micronuclei (MN) in peripheral blood lymphocyte. The concentration of As in drinking water from Socaire was 260 µg·L^−1^ and results showed a significant increase in the frequency of MN among the exposed groups, in agreement with similar bio-monitoring studies conducted by other authors on individuals who are environmentally exposed to arsenic through drinking water.

In a study conducted in Chile on a population exposed to As through drinking water, As levels were found to increase by about 30% in urine for t-As after DMPS [46]. In another study conducted by Pandey *et al*. [26], a clear correlation was established between clinically observable symptoms, urine As levels, and As intake via drinking water. The intensive study on urinary As levels confirms the pronounced chronic exposure these people have had to As. In the affected area, substituting the contaminated source for less contaminated sources has resulted in lower levels of urinary As. This indicates that arsenic exposure has become a greater concern than ever before in terms of its potential adverse health effects.

In our study the Pearson correlation analysis indicated that the association between a very high As intake in drinking water and food As levels in urine is not significant. This result is in agreement with those determined by Uchino *et al*. [47] who used As concentration in tubewell water and food composites (mainly vegetables and cereals) to establish whether there is any correlation between As dietary intake and As concentrations in hair and urine. They observed a correlation between As concentration in hair and dietary intake (*R*^2^ = 0.45, *p* < 0.001) and a weaker correlation between urine and dietary intake (*R*^2^ = 0.13, *p* < 0.001). The authors, however, do not explain these results. Whereas, our results could be explained due the existence of differences in the response to As among individuals within the same population. Such differences in individual susceptibility given the same exposure conditions could be due to differences in As metabolism [48]. Variations in urinary As excretion profiles among individuals from relatively homogenously exposed populations have been previously reported [10,21,49] providing further evidence of inter-individual variation in As metabolism.

Our study is limited by its small sample size, which makes it difficult to test the reliability of the observed variability in As metabolism. Hopenhayn-Rich *et al.* [50] studied a large number of subjects in the same geographic area of Chile and inter-individual variation was observed within the population.

The influence of some biological factors such as gender, age, health-status parameters, nutrition and various lifestyle characteristics on chronic As-toxicity has ben researched previously. This research has revealed that urinary As levels were higher in men than women, and increased with age [49]. These findings are consistent with our results: individual 20 (who had high As levels) was a 68-year-old man, whereas individual 1 (lowest As levels) is a 39-year-old woman (Table 1). However, it is not possible to ensure that these biological factors affected the total urinary As levels in our work.

The influence of occupational exposure to As (mining) does not seem to affect either health status or As urinary levels (Table 1 and Table 5). Individuals 16 and 20 do not show a clear effect on either health status or arsenic urinary levels due mining activities.

Limitations of this study include potential selection bias, as participants were mostly adult females. This could be addressed in future studies conducted in Chile by recruiting adult males outside of typical work hours, during the evening and on weekends.

## 5. Conclusions

All of the interviewed participants exceeded the FAO/OMS reference intake (149.8 μg of i-As·day^−1^) by approximately nine times. The results show that drinking water and food substantially contribute to As intake and increased exposure risk for adults in contaminated areas, as demonstrated by the very high As levels in urine, indicating that this contribution must be considered when interpreting dose-response modeling based on the use of available epidemiological data. Further work is required to characterize exposure in residents known to be exposed to high concentrations of environmental As.

## Abbreviations

As: Arsenic
i-As: Inorganic arsenic
t-As: Total arsenic
QA/QC: Quality Analytic/Quality Control analysis of the analytical data
PTWI: Provisional Tolerable Weekly Intake
TDI: Tolerable Daily Intake
MN: Frequency of Micronuclei
BNMN: Frequency of Binucleated Micronucleated Cells
WHO: World Health Organization
FAO: Food Agricultural Organization
JECFA: Joint Expert Committee for Food Additives
DMPS: Dimercapto-1-Propane Sulfonate
